# Complications of Small Aperture Intracorneal Inlays: A Literature Review

**DOI:** 10.3390/life13020312

**Published:** 2023-01-22

**Authors:** María Carmen Sánchez-González, Estanislao Gutiérrez-Sánchez, José-María Sánchez-González, Concepción De-Hita-Cantalejo, Ana-María Pinero-Rodríguez, Timoteo González-Cruces, Raúl Capote-Puente

**Affiliations:** 1 Department of Physics of Condensed Matter, Optics Area, University of Seville, 41012 Seville, Spain; 2 Department of Surgery, Ophthalmology Area, University of Seville, 41009 Seville, Spain; 3 Department of Anterior Segment, Cornea and Refractive Surgery, Hospital La Arruzafa, 14012 Cordoba, Spain

**Keywords:** small aperture intracorneal inlay, SAICI, KAMRA inlay, corneal inlay pinhole, intraoperative complications, postoperative complications, refractive surgery, presbyopia

## Abstract

Presbyopia can be defined as the refractive state of the eye in which, due to a physiological decrease in the ability to accommodate, it is not possible to sustain vision without fatigue in a prolonged manner, along with difficulty focusing near vision. It is estimated that its prevalence in 2030 will be approximately 2.1 billion people. Corneal inlays are an alternative in the correction of presbyopia. They are implanted beneath a laser-assisted in situ keratomileusis (LASIK) flap or in a pocket in the center of the cornea of the non-dominant eye. The purpose of this review is to provide information about intraoperative and postoperative KAMRA inlay complications in the available scientific literature. A search was conducted on PubMed, Web of Science, and Scopus with the following search strategy: ("KAMRA inlay" OR “KAMRA” OR “corneal inlay pinhole” OR “pinhole effect intracorneal” OR “SAICI” OR “small aperture intracorneal inlay”) AND (“complication” OR “explantation” OR “explanted” OR “retired”). The bibliography consulted shows that the insertion of a KAMRA inlay is an effective procedure that improves near vision with a slight decrease in distance vision. However, postoperative complications such as corneal fibrosis, epithelial iron deposits, and stromal haze are described.

## 1. Introduction

Presbyopia can be defined as the refractive state of the eye in which, due to a physiological decrease in the ability to accommodate, it is not possible to sustain vision without fatigue in a prolonged manner, along with difficulty focusing on near vision [1,2,3,4]. Presbyopia depends not only on biological age but also on predominant factors such as visual defects and working distance and factors such as low light or fatigue disorders at the end of the day [5,6]. It is estimated that its prevalence in 2030 will be approximately 2.1 billion people [7].

Surgery for presbyopia includes a wide range of surgical approaches and procedures. Strategies include different corneal approaches, such as the application of excimer and femtosecond lasers in techniques such as monovision, sectoral modification in corneal multifocality (PresbyLASIK), and conductive keratoplasty, where electromagnetic radiation is used as the radiofrequency energy [8,9,10]. Similarly, corneal modifications assisted by femtosecond lasers such as IntraCOR and SUPRACOR are successfully used, where changes in the patterns of corneal aberrations are generated [11,12,13].

Currently, multifocal intraocular lenses (MIOLs) are also an effective alternative for the correction of pseudophakic presbyopia. MIOLs are grouped according to their optical characteristics: nonapodized (AcrySoft**^®^** IQ PanOptix**^®^**, Alcon Laboratories, Inc., Fort Worth, TX, USA) and apodized diffractive designs (ReSTOR**^®^**, Alcon Laboratories, Inc., Fort Worth, TX, USA), and refractive (Rezoom^TM^, AMO, Inc., Santa Ana, CA, USA), pseudo accommodative AcrySoft ReSTOR (Alcon Laboratories, Inc., Fort Worth, TX, USA), and extended depth of focus (EDOF) designs (Eden, SAV-IOLS.A., Neuchâtel, Switzerland) [14,15,16]. Additionally, correction of the multifocal design of the anterior chamber (Vivarte**^®^** Presbyopic, IOLtech, La Rochelle, France) with phakic intraocular lenses (pIOLs) is used for its predictability, fixation technique, and support for presbyopia correction [17,18].

KAMRA inlay surgery is a type of vision correction procedure that involves the implantation of a small, circular device called a KAMRA inlay into the cornea of the eye [19]. The KAMRA inlay is designed to help improve near vision in people with presbyopia, a condition that causes the loss of the eye’s ability to focus on close objects as we age [20]. The KAMRA inlay is made of a thin circular disk of polymer material that is just a few millimeters in diameter. It is placed in a layer of the cornea called the stroma, which is located just beneath the outermost layer of the cornea, called the epithelium [21]. The inlay is placed in the non-dominant eye, which is typically the left eye for right-handed individuals. The KAMRA inlay works by creating a small, circular opening in the center of the cornea that allows light to pass through and focus on the retina at the back of the eye [22]. This opening, known as a pinhole, narrows the focus of light entering the eye and improves the eye’s ability to see objects at close range. By using the “depth of focus” principle commonly used in photography, the KAMRA implant controls light transmission, allowing only focused light rays to reach the retina through a fixed 1.6 mm aperture. The inlay is designed to be small enough to be barely noticeable, but still large enough to allow a sufficient amount of light to pass through and improve near vision [23].

KAMRA inlay surgery is typically performed as an outpatient procedure and takes about 15–30 min to complete. The procedure is generally well tolerated and has a low risk of complications [24]. Before the procedure, the surgeon will numb the eye with anesthetic drops to reduce any discomfort during the procedure. The surgeon will then create a small flap in the cornea using a laser or a blade and lift it up to access the stroma [25]. Generally, KAMRA inlays are implanted mechanically or assisted by a femtosecond laser in people with presbyopia who do not have refractive errors. For people with refractive errors, a combined procedure, such as LASIK, can be performed to fix the refractive error at the same time as the inlay is implanted [26]. The KAMRA inlay is then carefully placed in the stroma and the corneal flap is replaced and sealed. After the procedure, the eye may be slightly red and swollen for a few days, and some people may experience mild discomfort or sensitivity to light [27]. These symptoms typically resolve on their own within a few days to a week. It is important for patients to follow the surgeon’s instructions for postoperative care, which may include using eye drops or ointment to keep the eye moist and prevent infection [28]. The surgeon will also schedule follow-up appointments to monitor the healing process and ensure that the inlay is functioning properly. Most people who have KAMRA inlay surgery experience a significant improvement in their near vision within a few days to a week after the procedure [29]. The results of the procedure are generally long-lasting, although the inlay may need to be replaced after several years if the patient’s vision begins to decline again [30]. KAMRA inlay surgery is an effective treatment option for people with presbyopia who want to improve their near vision without the need for glasses or contacts. It is generally a safe and well-tolerated procedure, with a low risk of complications. However, as with any surgical procedure, it is important for patients to carefully consider the potential risks and benefits and discuss them with their surgeon before deciding whether KAMRA inlay surgery is the right choice for them [27]. The approximate number of KAMRA inlays that have been implanted worldwide is 20.000 [31].

## 2. Corneal Inlay

Synthetic keratophakia was first described by Barraquer in 1949 [19]. However, the materials he used, i.e., flint glass and plexiglass, were found to be unsuitable due to biocompatibility issues. In 1960, other more transparent and permeable materials, hydrogel polymers, were tested to favor metabolic gradients through the stroma so that waste products pass into the aqueous humor and the flow of nutrients to the cornea is maintained [19].

There have been important advances in the design and material of corneal implants. Currently, the models used are thinner and permeable to oxygen, and the use of the femtosecond laser facilitates their intracorneal placement. Three types of implants are considered to have good results (Table 1): corneal reshaping inlays, Raindrop^®^ Near Vision (ReVision Optics, Lake Forest, CA, USA) [32]; refractive inlays, Flexivue Microlens™ (Presbia Coöperatief U.A., Irvine, CA, USA) [27] and Icolens System™ (Neoptics AG, Hünenberg, Switzerland) [27]; and small aperture intracorneal inlays, KAMRA™ (AcuFocus Inc., Irvine, CA, USA) [20]. The Raindrop inlay was discontinued in 2018, just 2 years after its FDA approval in 2016, due to corneal haze, while Flexivue Microlens has been awaiting FDA approval in the United States since 2019 [33].

The purpose of this review is to provide information about intraoperative and postoperative KAMRA inlay complications in the available scientific literature.

## 3. Literature Review

### 3.1. Search Strategy

A systematic literature search was performed using the PubMed/MEDLINE (77 articles), Web of Science (55 articles), and Scopus (54 articles) databases. The search strategy included the terms ("KAMRA inlay" OR “KAMRA” OR “corneal inlay pinhole” OR “pinhole effect intracorneal” OR “SAICI” OR “small aperture intracorneal inlay”) AND (“complication” OR “explantation” OR “explanted” OR “retired”). Databases were searched for publications from January 2011 to January 2022.

The inclusion criteria were as follows: (1) studies with humans; (2) case reports; (3) case series; (4) cohort, cross-sectional, and case–control studies; and (5) randomized clinical trials. The exclusion criteria were as follows: (1) animal studies; (2) the article was a letter to the editor, conference abstract, study protocol, or literary review; (3) the article was not available in English; and (4) nonindexed publications.

A total of 186 articles were identified. After removing duplicates, article grading and data extraction were independently performed by two authors, MCSG and RCP, according to the inclusion and exclusion criteria. If there was a conflict with the selection of an article, the third author, JMSG, decided the outcome. A total of 20 articles were finally included in the review.

### 3.2. Results of Literature Search

The literature consulted shows that both the insertion of a KAMRA in presbyopic emmetropes [29,34,35,36,37,38,39] and insertion combined with LASIK [40,41,42,43,44,45] or PRK [46] in patients with ametropia, in addition to the insertion in presbyopic phakic patients [47], is an effective procedure that improves near vision with a slight decrease in distance vision in the eye with the implant or in both eyes.

Dexl et al. [37] followed up with 32 patients for five years. Their results showed a significant improvement in uncorrected near visual acuity (UNVA) after one year that remained stable up to month 36. At 60 months, UNVA decreased slightly. Similarly, this also occurred with uncorrected intermediate visual acuity (UIVA), which improved from 20/32 to 20/25 at 12 months, remained stable up to 36 months, and at 60 months decreased slightly to 20/32.

However, postoperative complications such as halos, dry eyes, and alterations in night vision have been described [42] and hyperopic regression has been described after a follow-up period [35,36,41,42,46]. There are authors who describe the appearance of corneal fibrosis [30,48], epithelial iron deposits [35], and keratocyte activation around the back surface of the inlay [49,50] as possible causes of haze that require explantation.

Although the KAMRA corneal inlay is a removable device, patients may experience residual corneal haze, hyperopic shift, and deficits in uncorrected distance visual acuity (UDVA) after explantation compared to pre-implantation UDVA [33].

Darian-Smith et al. retrospectively analyzed the visual outcomes of KAMRA inlay insertion in a cohort of patients reporting success of procedure, complications, patient satisfaction, and refractive outcomes at the TLC Laser Centre, Toronto. The explantation rate was 11.42%; 28.5% of patients required enhancements after inlay insertion [51]. Moshirfar et al. evaluated 10 years of KAMRA corneal inlay explantation and associated visual outcomes. KAMRA explantation rate was 8.2% across 10 years in Salt Lake City, Utah, USA [33].

### 3.3. Complications

#### 3.3.1. Distance Visual Acuity

Although patients experience better near and intermediate vision after KAMRA surgery [51], follow-up with patients after implantation suggests decreased distance visual acuity in the implanted eye or in both eyes. Dexl et al. [35] reported a decrease in distance visual acuity with correction (CDVA) in emmetropic presbyopic patients. Additionally, Tomita et al. [44] and Vukich et al. [36] described how visual acuity decreased with distance in a group of post-LASIK and emmetropic patients after KAMRA implantation.

#### 3.3.2. Refractive Changes

Refractive instability is also a predictable consequence after KAMRA surgery and has been reported by several authors [41,47,52]. Moshirfar et al. [53], in a case series of 50 patients, reported keratometric and topographic changes that led to a change in refraction. At 3 years, 54% of the eyes implanted with a KAMRA had a hyperopic manifest refractive spherical equivalent (MRSE), 40% were myopic with respect to the initial value, and the mean keratometry (K_m_) was significantly increased at all postoperative measurements compared with baseline. Optimal near-vision results require slightly myopic MRSE, with −0.75 considered an ideal compromise between near and far vision.

#### 3.3.3. Decentration

KAMRA inlay implantation has been described as successful in the majority of patients; in the actual scientific literature the decentration and repositioning rate ranges from 1.2% to 8.8% [33]. The small-aperture corneal inlay implantation technique creates an intrastromal pocket with a femtosecond laser and the pinhole device is later placed into this pocket via a small incision [53]. The criteria for KAMRA inlay placement are targeted in the center of the Purkinje reflex. In cases where the Purkinje reflex and the pupil are separated by a few microns, the KAMRA inlay needs to be placed between the Purkinje reflex and the pupil [48]. The exact placement of the pinhole inlay is necessary to achieve good visual and refractive results.

**Figure 1:**
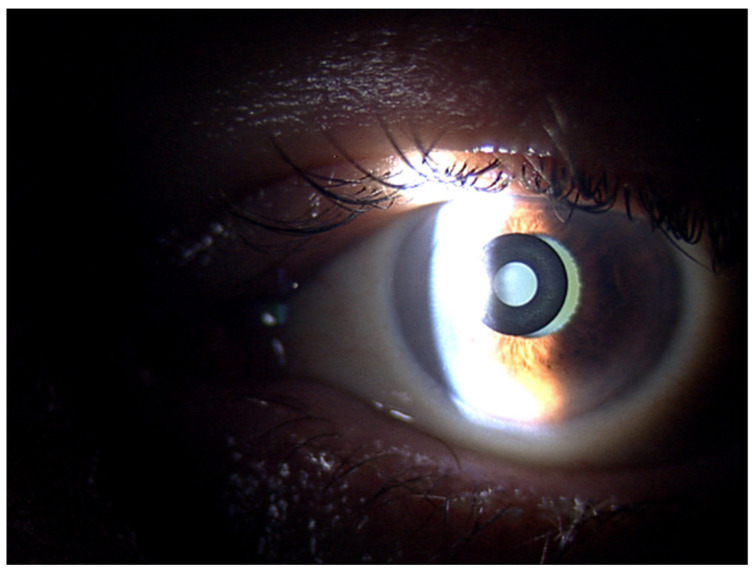
Slit-lamp examination. Decentration of a KAMRA inlay toward nasal area. A temporary area of the cornea free of the KAMRA is observed, which allows light to pass through.

Decentration of the inlay implies damage to visual quality and poor refractive results [54]. In Figure 1, a nasal decentration of the KAMRA inlay observed using the section illumination of a biomicroscope is presented. Repositioning should be performed after implantation to achieve good refractive results and improve near, intermediate, and distance vision. It is important to note that the KAMRA inlay is placed while the patient is lying down and the effect of gravity pushes down on the KAMRA inlay, which could increase the decentration of the device in the intrastromal pocket [52].

**Figure 2:**
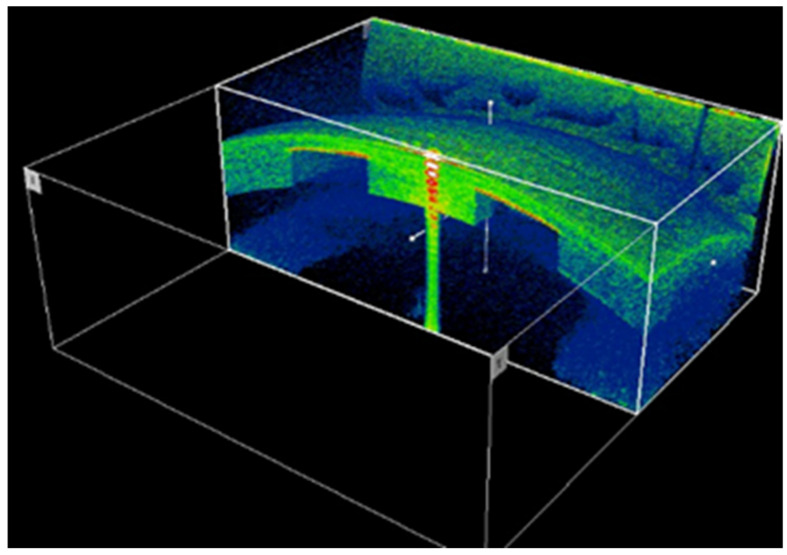
SD-AS-OCT of the anterior segment, revealing nasal decentration of the small-aperture corneal inlay. From left to right the nasal and temporal images are observed.

**Figure 3:**
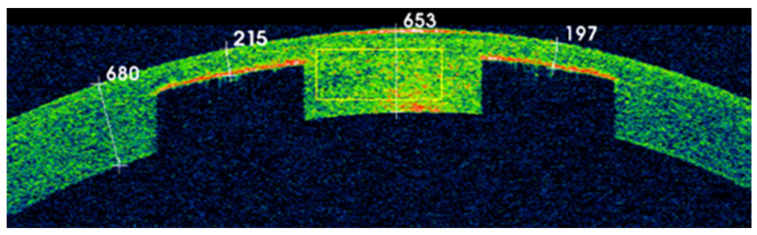
SD-AS-OCT of the anterior segment revealing a stromal hyperreflective signal. Caliper measurements from left to right (in µm): Total peripheral pachymetry (680), nasal KAMRA pocket depth (215), total central pachymetry (653), temporal KAMRA pocket depth (197).

New techniques and devices, such as ocular coherence tomography for anterior segment (AS-OCT) imaging, could help to locate and measure the decentration of a KAMRA inlay [50]. Figure 2 shows how AS-OCT technology reveals in a 3D-cube image how the inlay is partially displaced to the nasal zone. In another point of view, the AS-OCT could show us how deep the inlay is placed. Figure 3 shows the hyperreflective surface of the KAMRA inlay with a yellow and red image. Furthermore, the AS-OCT caliper could determine the depth of the inlay or the distance between the center of the pupil and the center of the inlay. This technology could be beneficial to perform after repositioning an inlay to move the pinhole device.

After the United States of America Food and Drug Administration approved KAMRA, the device gained in popularity [55]. The refractive results of this presbyopia treatment depend in large part on the centering of the pinhole device. Decentration of the KAMRA is principally due to the initial surgeon mispositioning it and inducing poor alignment [54]. This decentration is an intraoperative complication, while a displacement after the surgery indicates a migration in the inlay, therefore, migration is a postoperative complication. This issue has an explanation: the creation of a small pocket combined with the rough posterior face of the pinhole inlay implies that the device increased the adherence to the stromal pocket [41]. However, there are other intrastromal inlays that decentered after placement. The hydrogel corneal inlay Raindrop decentered after a corneal flap creation combined with a steroid that increased the intraocular pressure of the eye. In addition, the stromal keratopathy created by the increase in intraocular pressure possibly created an accumulation of fluid and a relocation of the hydrogel inlay [56].

#### 3.3.4. Migration and Extrusion

Migration and extrusion are two potential complications that can occur with the KAMRA inlay [24,28,40,49,52]. Migration refers to the movement of the inlay within the cornea after it has been implanted. This can occur if the inlay becomes dislodged from its intended position within the cornea. Extrusion refers to the complete removal of the inlay from the cornea [30,41,57]. Both migration and extrusion can occur as a result of improper implantation, infection, inflammation, or other factors. Symptoms of migration or extrusion may include blurred vision, eye irritation, and discomfort. In severe cases, migration or extrusion of the KAMRA inlay may require surgical intervention to remove the device and restore vision [24,28,40,49,52].

The permeability of implants to water and other molecules (especially glucose) determines the biocompatibility of the implant and its survival in the corneal stroma [56,58]. Extrusion is usually preceded by stromal necrosis. Initially, inlays were designed of poly (methyl methacrylate) and polysulfone. The low permeability of these materials caused inadequate corneal nutrition, inducing thinning of the anterior stroma and keratolysis [19]. Hydrogel implants with variable water content have shown good results and absence of long-term extrusion.

#### 3.3.5. Stromal Haze

**Figure 4:**
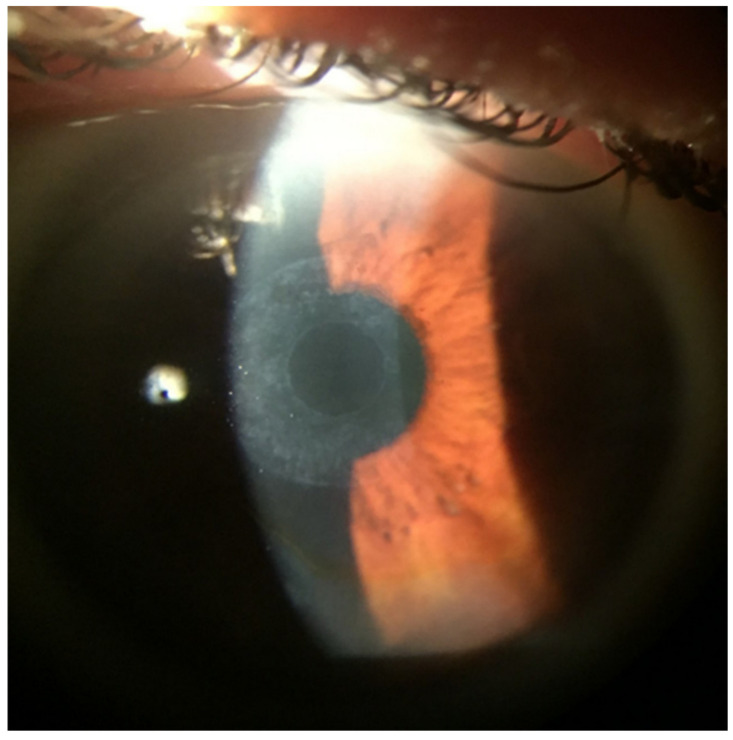
Slit-lamp examination at 2 months after removal. Stromal leukoma-shaped 360 degree ring (stromal footprint) associated with the KAMRA and corneal epithelial iron deposits in a half-moon shape (similar to a Fleischer ring).

Corneal haze (Figure 4) is a common complication of KAMRA inlay surgery, although it is typically mild and does not cause significant vision loss. Corneal haze is a condition in which the cornea becomes cloudy or hazy, which can interfere with vision [27]. It is caused by the formation of scar tissue in the cornea as a result of the surgical procedure [25]. Symptoms of corneal haze may include blurred or hazy vision, glare or halos around lights, and sensitivity to light. These symptoms may be more noticeable at night or in low light conditions. In most cases, corneal haze is mild and does not cause significant vision loss, but it can sometimes interfere with activities such as driving or reading [59,60]. Treatment for corneal haze typically involves the use of eye drops or ointments to reduce inflammation and promote healing. In some cases, laser treatment may be necessary to remove the scar tissue and improve vision [30,48]. Most people with corneal haze experience improvement in their vision within a few weeks to a few months after treatment, although it may take longer for some people. It is important for people who have had KAMRA inlay surgery to be aware of the potential for corneal haze and to monitor their vision closely after the procedure. Overall, while corneal haze is a potential complication of KAMRA inlay surgery, it is generally mild and can be effectively treated with the use of eye drops or ointments and, in some cases, laser treatment [27,40,48].

The migration of surface proinflammatory cytokines is a local source of proteolytic enzymes and cytokines responsible for interface haze formation [57,61]. It is a predictable complication in response to the implantation of synthetic materials [61]. The greater the depth of the inlay implantation, the lower the keratocytic activation and development of haze [41]. Optical coherence tomography (OCT) is very effective in guiding the depth of femtosecond laser pocket creation for inlay implantation.

The cornea is an avascular tissue: on its anterior face, in intimate contact with the precorneal tear film, it absorbs oxygen, and on its posterior face, bathed by aqueous humor by diffusion, it receives glucose.

Keratocytes occupy 3–5% of the stromal volume. Its function consists of maintaining the collagen fibers and the extracellular matrix through a constant synthesis activity favoring metabolic gradients through the stroma so that waste products pass into the aqueous humor and nutrients flow to the cornea [62].

The microperforations of the KAMRA inlay optimize the flow of nutrients from the cornea [63]. However, the small-aperture corneal inlay can disrupt the exchange of oxygen and glucose. The result is the release of growth factors and proinflammatory cytokines that can generate myofibroblasts and be the cause of persistent fibrosis [30,48].

Numerous authors describe a series of cases of stromal opacity after implantation of a KAMRA [38,40,45,60]. The treatment of severe opacity includes the use of topically applied drugs. Corticosteroids and mitomycin C are usually effective. If the opacity persists over time, implant explantation is recommended [50].

#### 3.3.6. Infectious Keratitis

Infectious keratitis is an infection of the cornea; it is a serious and potentially vision-threatening condition that can occur after KAMRA inlay surgery, although it is rare [48]. Infectious keratitis is caused by bacteria, fungi, or viruses that enter the eye and infect the cornea. It can occur as a result of a variety of factors, including trauma to the eye, contact lens wear, and surgery [26,64]. KAMRA inlay surgery involves the creation of a small flap in the cornea, which can increase the risk of infection if the eye is not properly cared for after the procedure. Symptoms of infectious keratitis may include redness, pain, and sensitivity to light in the affected eye, as well as discharge around the eye [26,64]. In some cases, the infection may cause the cornea to become cloudy or hazy, which can interfere with vision. Treatment for infectious keratitis typically involves the use of antibiotics or antifungal medications to clear the infection and prevent further damage to the eye. In severe cases, surgery may be necessary to remove the infected tissue and prevent the spread of the infection [28,48].

Duignan et al. [28] describe a series of cases that, after implantation, presented anterior-chamber cellular reactions, epithelial defects, conjunctival hyperemia, and corneal infiltrates characteristic of infectious keratitis. The patients were treated with antibiotics. Bouheraoua et al. [65] described the case of a woman with epithelial growth around an implant to correct hyperopia (Permavision, Anamed Inc., Lake Forest, CA, USA). It was probably the cause of the infectious keratitis that appeared seven years after the surgery.

#### 3.3.7. Epithelial Ingrowth

Epithelial ingrowth is a potential complication that can occur after the implantation of a KAMRA inlay [61]. Epithelial ingrowth occurs when cells from the epithelium, the outer layer of the cornea, grow into the area surrounding the inlay [26]. Due to poor pocket adhesion or the presence of epithelial foreign bodies, cells migrate from the stromal pocket edge toward the pocket–stromal interface [26]. This can cause the inlay to become dislodged or distorted, leading to reduced vision and other symptoms [59]. Epithelial ingrowth may also cause inflammation and scarring, which can further compromise vision [66]. Epithelial ingrowth is more likely to occur if the inlay is not properly implanted or if there is damage to the cornea during the implantation procedure. It can also occur as a result of infection or inflammation. Symptoms of epithelial ingrowth may include blurred vision, eye irritation, and discomfort. In severe cases, surgical intervention may be necessary to remove the inlay and restore vision [61].

Dexl et al. [35] described a case of epithelial growth in a series of 32 cases with a KAMRA inlay. The patient presented epithelial growth after lifting the flap for the treatment of postoperative striae. Rafic et al. [59] describe epithelial growth in a 52-year-old man with basement membrane dystrophy after undergoing a combination of hyperopic laser keratomileusis and KAMRA implantation. Most likely, epithelial growth could be the cause of the basement membrane dystrophy that could have leaked proinflammatory cytokines and chemokines to the stromal interface and amplified keratocyte activation.

#### 3.3.8. Binocular Vision

Castro et al. [67] showed a deterioration of binocular vision in a group of patients who underwent a simulation of KAMRA inlay. Anisocoria was induced with a contact lens that has a partially opaque peripheral area. The lens was placed on the nondominant eye. Measurements were made under two conditions: induced anisocoria and induced anisocoria combined with monovision using two additional powers inserted in a trial frame: +0.75 and +1.25. Stereoacuity was performed at three distances: near, intermediate, and far. The study showed a deterioration in stereoacuity in all induced anisocoria conditions. This deterioration was significant at intermediate and close distances. Additionally, Lin et al. [68] reported a deterioration in stereo acuity after KAMRA surgery.

Binocularity is the result of the processing of motor and sensory skills that allows obtaining a spatial reference from using both eyes simultaneously, merging a single image at the cortical level; when this happens, a stereoscopic vision or three-dimensional image is achieved [69,70,71,72]. Any type of alteration in binocular vision will establish a lack of brain integration and interpretation.

## 4. Conclusions

KAMRA inlay surgeries in presbyopic emmetropes and KAMRA inlay surgeries combined with LASIK in patients with ametropia is an effective procedure that improves uncorrected near visual acuity with a slight decrease in distance vision in the eye with the implant or in both eyes.

This pinhole implant surgery can have moderate to severe complications, such as refractive instability, epithelial ingrowth, or infectious keratitis. Refractive outcomes should be considered with caution due to possible conflicts of interest between the authors of the publications and manufacturers.

## Figures and Tables

**Table 1:** Characteristics of corneal inlays.

Corneal Inlay	Name	Diameter (mm)	Thickness (µm)	Material	Placement (Flap/Pocket) µm
Reshaping	Raindrop^®^	1.5–2	Periphery (10) Center (30)	HAH	130–150
Refractive	Flexivue Microlens™	3,2	15–20	HAH with ultraviolet filter (Contaflex C126)	280–300
	Icolens™	3	15–20		
SAICI	KAMRA™	3,8 1.6 (Pinhole)	5	PFC	200–250

SAICI: small aperture intracorneal inlay; HAH: hydrophilic acrylic hydrogel; PFC: polyvinylidene fluoride and carbon.
